# *Artemisia scoparia* extract inhibits oxidative stress and ferroptosis to ameliorate MASH through AGE-RAGE and JAK1-STAT3 signaling

**DOI:** 10.3389/fphar.2026.1792221

**Published:** 2026-04-15

**Authors:** Linlin Wang, Yu Miao, Hailong Wang, Hamulati Hasimu, Tengfei Ji, Hua Huang, Guanhua Du, Haji Akber Aisa, Xuelei Xin

**Affiliations:** 1 State Key Laboratory Basis of Xinjiang Indigenous Medicinal Plants Resource Utilization, and the Key Laboratory of Chemistry of Plant Resources in Arid Regions Xinjiang Technical Institute of Physics and Chemistry, Chinese Academy of Sciences, Urumqi, China; 2 Xinjiang Key Laboratory of Uygur Medicine, Xinjiang Institute of Materia Medica, Urumqi, China; 3 University of Chinese Academy of Sciences, Beijing, China; 4 Xinjiang Medical University, Urumqi, China; 5 State Key Laboratory of Bioactive Substance and Function of Natural Medicines, Institute of Materia Medica, Chinese Academy of Medical Sciences and Peking Union Medical College and Beijing Key Laboratory of Drug Target and Screening Research, Beijing, China

**Keywords:** AGE–RAGE signaling pathway, *Artemisia scoparia*, ferroptosis, JAK–STAT signaling pathway, metabolic dysfunction-associated steatohepatitis, metabolomics, transcriptomics

## Abstract

**Background:**

Metabolic dysfunction-associated steatohepatitis (MASH) is the most prevalent chronic liver disease worldwide; however, few effective therapeutic options are available for MASH. *Artemisia scoparia* is a medicinal plant that has been widely utilized in traditional medicine to treat liver-related ailments. Nonetheless, the effects and underlying mechanisms of *A. scoparia* in the context of MASH remain poorly understood.

**Aim of the study:**

The objective of this research was to assess the protective effects and further mechanisms of *A. scoparia* extract (AS) on a MASH mice model.

**Methods:**

The protective effects of AS were evaluated both *in vivo* and *in vitro*, with the therapeutic efficacy of AS being characterized through the detection of biochemical markers, histological analysis, and Oil red O staining. To elucidate the underlying mechanisms and pharmacodynamic basis of AS, a comprehensive set of techniques were applied, including transcriptomics, metabolomics, Western blotting, and immunofluorescence staining.

**Results:**

AS reduced the blood lipid indices and inflammatory levels in the MASH mouse model and decreased lipid droplet accumulation in FFA-induced HepG2 cells. Transcriptomic and metabolomic analyses indicated that AS regulates 30 dysregulated genes (e.g., *Gm15622, Pdia6,* and *Derl3*) and controls 60 metabolic metabolites (e.g., heptadecanoic acid, 5b-cyprinol sulfate, and taurodeoxycholic acid) to ultimately affect core pathways involved in lipid metabolism and inflammation. Furthermore, AS was proven to exert a hepatoprotective effect by inhibiting inflammation and ferroptosis, along with weakening the advanced glycation end product–receptor for advanced glycation end products (AGE–RAGE) pathway and the Janus kinase–signal transducer and activator of transcription (JAK–STAT) pathway *in vivo* and *in vitro*.

**Conclusion:**

This study first elucidates the mechanism through which AS ameliorates MASH through integrated multi-omics analysis, providing experimental evidence for further development of natural therapeutic agents.

## 1 Introduction

Metabolic dysfunction-associated steatohepatitis (MASH) is a complex metabolic-related fatty liver disease characterized by steatosis and lipid accumulation in the hepatocytes, accompanied by lipotoxicity and inflammatory damage to hepatocytes (Tincopa et al., 2024). MASH increases the risk of complications from hepatic decompensation and is linked to cirrhosis and liver cancer (Wu et al., 2024). There has been a steady rise in MASH cases, with reports indicating that its global prevalence may reach as high as 20%, representing a considerable health threat to society and placing a substantial economic strain (Pan et al., 2025). Nevertheless, the therapeutic strategies for MASH remain unsatisfactory; therefore, finding new innovative therapeutic drugs for MASH is an urgent necessity.

Abnormalities in lipid metabolism and inflammation are the primary factors that contribute to MASH (Lee et al., 2023). A sustained high-fat dietary (HFD) intake leads to an increased release of free fatty acids from adipose tissue, which, in turn, disrupts the balance of lipid metabolism in the liver. This imbalance promotes oxidative damage to the liver, initiates an inflammatory response, and encourages fibrosis, ultimately escalating the development of MASH from simple hepatic steatosis to significant liver damage (Lee et al., 2023). The inactivation of NFATc4 diminishes lipid accumulation and the inflammatory response in the liver, thus mitigating the effects of MASH (Du et al., 2020). This discovery advanced methods for the prevention and treatment of MASH by providing a paradigm for understanding the molecular mechanisms governing hepatic lipid metabolism and inflammation. Consequently, strategies that aim at inhibiting abnormal lipid metabolism and curbing inflammation might provide personalized approaches for MASH prevention and treatment, which represents a significant avenue for the innovation of novel therapeutics against MASH.

*Artemisia scoparia* Waldst. and Kit. (Asteraceae), known in traditional Chinese medicine as “Yinchen” (*Artemisiae Scopariae Herba*), is a botanical drug that is officially documented in the Chinese Pharmacopoeia. In traditional medicine, it is often utilized for the management of jaundice (characterized by dampness–heat in the liver and gallbladder) (Ding et al., 2021), hepatobiliary disorders (including hepatitis and cholecystitis) (Wei et al., 2022), and dyspepsia (Boudreau et al., 2021). Pharmacological research has shown that *A*. *scoparia* possesses a spectrum of pharmacological properties, including antitumor, antibacterial, antiviral, anti-inflammatory, antioxidant, and hepatoprotective properties (Ding et al., 2021). *A. scoparia* extract (AS) attenuates non-alcoholic fatty liver disease in mice fed a HFD by enhancing hepatic insulin and AMPK signaling (Wang et al., 2013). Nevertheless, the specific therapeutic impacts of *A*. *scoparia* on MASH, along with their mechanisms, have yet to be clarified *in vivo.*

In recent years, there have been significant advancements in metabolomics and transcriptomics, showcasing their effectiveness in predicting targets and analyzing comprehensive mechanisms. These methodologies have been widely utilized in researching the pathogenesis of diverse diseases and in the development of pharmaceuticals (Deng et al., 2024; Ge et al., 2025). By quantitatively analyzing small-molecule metabolites found in organisms, metabolomics has established itself as a crucial link between traditional Chinese medicine and modern medical practices. Transcriptomics enables a systematic examination of gene expression variations that are associated with disease progression, influenced by different preparations of traditional Chinese medicine. Additionally, the integration of both these complementary technologies transcends the limitations that are inherent in single-omics approaches, facilitating a more thorough and detailed investigation of the molecular mechanisms underlying biological systems (Wang Y. et al., 2025).

The current treatment of MASH is based on a comprehensive strategy of “lifestyle intervention as the foundation and drug treatment as a supplement.” Lifestyle interventions include a low-calorie diet, regular exercise, and weight control, which are the basic means to slow down the progression of the disease; however, patients’ compliance is poor, and the long-term intervention effect is limited (Rinella et al., 2023). In March 2024, the U.S. FDA approved resmetirom (trade name: Rezdiffra), the first specific drug approved especially for the treatment of MASH, fulfilling the long-term gap of having no specific drugs in this field. As a selective thyroid hormone receptor β (THR-β) agonist, resmetirom is currently only approved for adult patients with non-cirrhotic MASH accompanied by moderate-to-advanced liver fibrosis (F2–F3 stage) [Resmetirom (MGL-3196) for the treatment of non-alcoholic steatohepatitis: a multi-center, randomized, double-blind, placebo-controlled, phase-2 trial, 2019]. In addition, there are limitations such as the high price of the original drug (approximately 420 yuan per tablet in the United States) and the need for improvement in long-term safety data (Petta et al., 2024; Harrison et al., 2024b). Therefore, the development of new anti-MASH drugs has a long way to go.

## 2 Materials and methods

### 2.1 Reagents and materials

#### 2.1.1 Preparation of *Artemisia scoparia* extract (AS)

*A. scoparia* plants were collected from the suburban areas of Urumqi City, Xinjiang, China. After botanical identification by Professor He Jiang from the Xinjiang Institute of Materia Medica, a voucher specimen (No. 20141114) was archived at the same institute. Dried plant matter (5 kg) was mechanically crushed, followed by three rounds of aqueous extraction (75 L of water, reflux extraction for 1 h each time). The combined filtrate was concentrated *in vacuo* at 60 °C. The concentrate was diluted with 10-fold volume of water and subjected to a D101 macroporous resin column. The D101 macroporous resin was pretreated for 12 h before use. After absorption, it was washed with water until the reducing sugar test was negative, followed by elution with 70% ethanol. The collected eluate fractions were concentrated under reduced pressure to a standardized density and then vacuum-dried at 60 °C to obtain 41 g of AS, corresponding to an extraction yield of 0.82%. This corresponds to a DER native of approximately 122:1. Furthermore, the chemical characterization of AS, including HPLC-UV (DAD) analysis and high-resolution mass spectrometry of its metabolites, has been reported previously. The detailed methodology and corresponding results are described in the Wang et al. (2018).

#### 2.1.2 Materials

Resmetirom was purchased from Manster Biotechnology (Chengdu, China). High-fat diet (HFD; containing 35% fat, 26% protein, and 26% carbohydrates by weight; H10060) was supplied by HFK Bioscience Co. Ltd. Alanine aminotransferase (ALT, 20230314), aspartate aminotransferase (AST, 20230308), triglycerides (TG, 020090), cholesterol (CHO, 020080), and high-density lipoprotein cholesterol (HDL-C, 20235) were supplied by BioSino Bio-Technology and Science Inc. (Beijing, China). Kits for interleukin-1β (IL-1β, CME0015) and tumor necrosis factor-α (TNF-α, CME0004) were sourced from 4A Biotech, which is based in Beijing, China. Additionally, the malondialdehyde (MDA, A003-1), superoxide dismutase (SOD, A001-3), glutathione (GSH, A006-1-1), and reactive oxygen species (ROS, E004-1-1) assay kits were procured from JianCheng Bioengineering Institute in Nanjing, China. The Oil red O staining kit (G1262) was purchased from Solarbio (Beijing, China). The primary antibodies used for immunofluorescence, namely, JAK1 (A18323), p-JAK1 (AF2012), STAT3 (9139T), and p-STAT3 (ab32143), were obtained from ABclonal (Wuhan, China), Affinity Biosciences (Jiangsu, China), and Abcam (Shanghai, China), respectively. STAT3 (9139T) and advanced glycation end product (AGE, C01328) were obtained from Cell Signaling Technology (Shanghai, China). Antibodies against Nrf2 (ab137550), HO-1 (ab13248), SLC7A11 (ab175186), GPX4 (ab125066), ACSL4 (ab155282), Alox15 (ab80495), FTH1(ab183781), and RAGE (ab216329) were obtained from Abcam (Cambridge, United Kingdom).

Peroxidase-labeled goat anti-rabbit IgG (BA1054), peroxidase-labeled goat anti-mouse IgG (BA1050), and RIPA lysis buffer (P0013B) were purchased from Boster Biological Technology (Wuhan, China).

HepG2 cells were kindly provided by Xinjiang Medical University. Oleic acid (112-80-1) and palmitic acid (ST3330-25 g) were purchased from Solarbio (Beijing, China) and Beyotime (Shanghai, China), respectively. The information of all protein antibodies is provided in the Supplementary Material.

### 2.2 Dose selection

The dose of AS (400 mg/kg) was determined through a preliminary dose–response study in HFD-fed mice, in which 200, 400, and 800 mg/kg doses were tested for their effects on serum ALT and liver histology. The 400-mg/kg dose showed optimal improvement in steatosis and inflammation without adverse effects. Moreover, preliminary experiments conducted in our laboratory demonstrated that similar doses of the AS extract were utilized in related literature, which provided a reference basis for the dose selection in this study (Wang et al., 2018). Resmetirom (12 mg/kg) was chosen based on its established treatment efficacy in murine NASH models (Harrison et al., 2023).

### 2.3 Animal treatment

A total of 40 male C57BL/6J mice (body weight: 18 g–22 g) were procured from Beijing HFK Biotechnology Co., Ltd. [License No. SYXK (Jing) 2019-0023]. The animals were maintained under specific pathogen-free (SPF) conditions and were housed five per cage with a 12-h/12-h light–dark cycle. Following a 7-day acclimatization period, they were randomly assigned to two cohorts: the normal control group (NC, n = 10) fed a standard chow diet and tap water *ad libitum* and the HFD group (n = 30) fed a HFD.

During weeks 1–8, the HFD group was maintained on the HFD to induce lipid accumulation. From week 9 to week 14, the HFD group was intraperitoneally injected with 1% CCL_4_ olive oil solution at a dose of 2 mL/kg body weight, twice weekly. Concurrently, throughout weeks 9–10, four mice were randomly selected from both the NC and HFD groups every week for tail-vein blood collection (50 µL), and serum levels of AST, ALT, TG, and total cholesterol (T-CHO) were measured to monitor the progression of liver injury. At the end of week 10, the MASH model was confirmed to be successfully established based on the serum biochemical indices.

At the onset of week 11, the HFD-induced mice were further randomized into three subgroups (n = 10 per subgroup): the model group (HFD-M), positive control group (resmetirom, 12 mg kg^-1^), and *A. scoparia* group (AS, 400 mg kg^-1^). All drugs were formulated as 0.5% CMC-Na suspensions and administered via oral gavage daily. Throughout the treatment period (weeks 11–15), the NC group were fed the standard diet, while the HFD-M, positive control, and AS groups were maintained on the HFD. The CCL_4_ injection was discontinued at the end of week 14.

After 5 weeks of continuous drug administration (at the end of week 15), all mice were fasted for 12 h with free access to water, followed by the collection of serum and liver samples for subsequent analysis. Notably, week 15 was selected as the study endpoint, which is a time point consistent with those in previous studies [1-2].

This study was approved by the Animal Ethics Committee of the Xinjiang Institute of Materia Medica (Approval No. XJIMM–20231106), and all procedures were performed in strict accordance with the Guidelines for the Care and Use of Laboratory Animals (Wang S.-J. et al., 2025; Cheng et al., 2025).

### 2.4 Biochemical analysis

Serum samples were collected and assayed for ALT, TG, CHO, AST, and HDL-C levels on an automatic biochemical analyzer (Toshiba Accute TBA-40FR, Tokyo, Japan).

### 2.5 IL-1β and TNF-α assay

The liver tissue of mice was homogenized and centrifuged for 10 min at 3,000 rpm min^-1^, and the resulting supernatant was assayed for IL-1β and TNF-α following the manufacturer’s guidelines.

### 2.6 Pathological assessment

Hepatic specimens were fixed in neutral buffered formalin for over 6 h, processed through a graded ethanol series for dehydration, and finally embedded in paraffin wax. For histological analysis, 10-μm slices were generated from the paraffin-embedded blocks. The sections were then subjected to HE, Oil red O, and Masson’s staining following established protocols. Finally, the stained sections were imaged with an optical microscope (Nikon Eclipse Ti, Japan).

### 2.7 Liver transcriptome sequencing and data analysis

Total RNA from liver tissues was purified with the TRIzol reagent. Subsequently, the RNA was quantified and purified to ensure high-quality samples that were appropriate for sequencing. The resulting RNA libraries were then prepared and profiled on the NovaSeq X Plus platform (Illumina, Inc., San Diego, CA, USA). After sequencing, differentially expressed genes (DEGs) were screened using the DESeq2 software (http://bioconductor.org/packages/stats/bioc/DESeq2/). The thresholds set for determining significant gene expression differences were an absolute log_2_ fold change (FC) of |log_2_ FC| ≥ 1 and a *p*-value of less than 0.05, which ensured that only DEGs with notable biological relevance were included in the analysis. To further elucidate the biological roles of the resulting DEGs, Gene Ontology (GO) and Kyoto Encyclopedia of Genes and Genomes (KEGG) enrichment analyses were conducted. These analyses were implemented based on GOATOOLS (https://github.com/tanghaibao/GOatools) in conjunction with the Python SciPy package (https://scipy.org/install/).

### 2.8 Metabolomics

#### 2.8.1 Extraction of liver tissue metabolites

Liver samples (approximately 50 ± 5 mg per mouse, n = 6 mice per group) were collected. Each individual sample was placed into a separate 2-mL centrifuge tube for independent sampling and detection. To facilitate the extraction of metabolites, 800 μL of a methanol–water extraction solution was introduced into each tube. Next, the samples were ground for a duration of 6 min at a temperature of −10 °C and a frequency of 50 Hz using a cryogenic tissue homogenizer. Following this grinding procedure, the samples underwent low-temperature ultrasonic extraction for 30 min at 4 °C and a frequency of 40 kHz. To finalize the extraction, the samples were incubated at −20 °C for 30 min and then centrifuged at 13,000 g for 15 min at 4 °C. The collected supernatant was ready for subsequent analysis. During data processing, intergroup comparisons were conducted on a group-wise basis, and the statistical data of all individual samples were presented for analysis.

#### 2.8.2 LC–MS/MS

Liver tissues were subjected to a thorough analysis utilizing the sophisticated UHPLC-Triple TOF 6600 system (AB SCIEX, USA). A 10-μL supernatant of the liver tissue sample was injected into an ACQUITY UPLC HSS T3 column (100 mm × 2.1 mm i. d., 1.8 μm; Waters, Milford, USA). The system was operated at a flow rate of 0.40 mL/min while maintaining a column temperature of 45 °C to ensure optimal separation of metabolites. The mobile phases used for the separation process were as follows: phase A comprised 95% water and 5% acetonitrile, while phase B comprised 47.5% acetonitrile, 47.5% isopropanol, and 5% water, with both phases supplemented with 0.1% formic acid to enhance ionization. The solvent gradient employed during the analysis was meticulously designed; it began with 0% B from 0 to 0.2 min, transitioned to 25% B from 0.2 to 3 min, increased from 25% B to 100% B from 3 to 9 min, was maintained at 100% B for 1 minute between 9 and 10 min, then rapidly returned to 0% B from 10 to 10.1 min, and finally maintained at 0% B from 10.1 to 12 min. Several critical parameters were set for this process, including an ion spray voltage of 5,500 V (positive) and −4,500 V (negative). The scanning range was established within 50 m/z–1,200 m/z, with the sheath gas flow configured at 50 arbitrary units, while the auxiliary heating gas flow was set to 13 arbitrary units. Furthermore, the temperature of the ion source was maintained at 450 °C, and the collision energy was modified to levels of 20, 40, and 60 V to promote efficient ion fragmentation for the analysis.

#### 2.8.3 Data analysis of metabolomics

The raw data were analyzed using Progenesis QI software from Waters Corporation, USA. Following this, the metabolites were identified by comparing the MS and MS/MS data with those of public databases such as HMDB and METLIN, along with a custom database created by Majorbio. The data matrix generated was subsequently transferred to the Majorbio analysis platform (cloud.majorbio.com) for further analysis. In the analysis pipeline, the first phase involved preprocessing the data matrix to prepare it for subsequent evaluations. Once the preprocessing was complete, PLS-DA and OPLS-DA were conducted using the ropls package, specifically version 1.6.2 in R software. To ensure the reliability of the models, their stability was assessed through a rigorous seven-fold cross-validation process. The identification of differential metabolites was based on two main statistical criteria derived from the OPLS-DA model: the variable importance in projection (VIP) values and the *p*-values obtained from Student’s t-test. Metabolites were classified as significant based on specific benchmarks: they had to demonstrate VIP values exceeding 1, in addition to *p*-values falling below the threshold of 0.05. For further biological insights, these differential metabolites were subjected to annotation via KEGG analysis. Specifically, pathway enrichment analysis was carried out utilizing the Python package “scipy.stats.” Additionally, Fisher’s exact test was applied to ascertain the biological pathways that were most relevant to the treatment applied in the experiment.

### 2.9 Immunofluorescence analysis

Immunofluorescence (IF) staining was performed on liver tissues to assess the expression and phosphorylation levels of JAK1 and STAT3. Briefly, consecutive tissue sections with a thickness of 5 μm were prepared and subjected to standard experimental procedures. The sections were first incubated with primary antibodies targeting JAK1, phosphorylated JAK1 (p-JAK1), STAT3, and phosphorylated STAT3 (p-STAT3) at 4 °C overnight. After subsequent incubation at the same temperature, the sections were treated with fluorescence-conjugated secondary antibodies. To visualize the cell nuclei, DAPI staining solution was applied and incubated for 10 min. Images of the stained sections were finally captured under a ×200 magnification field using a Nikon Ni-E microscopy imaging system.

### 2.10 HepG2 cell culture

HepG2 cells were maintained in DMEM containing 10% FBS, 100 U/mL penicillin, and 100 μg/mL streptomycin at 37 °C with 5% CO_2_. Cells were passaged for further study when they reached 80% confluence.

### 2.11 Preparation of free fatty acids (FFAs)

Oleic acid (OA) and palmitic acid (PA) [0.342 g of oleic acid and 0.170 g, respectively] were mixed at a ratio of 2:1 (OA: PA). The mixture was dissolved in isopropanol to a final volume of 10 mL and ultrasonicated at 50 °C for 10 min. The solution thus obtained was sterilized by filtration through a 0.22-μm microporous membrane under aseptic conditions to obtain a 200-mM FFA stock solution. This stock solution was subsequently diluted with complete culture medium to a working concentration of 10 mM for subsequent experiments.

### 2.12 *In vitro* drug intervention

For the *in vitro* model, a blank control group, a model group (0.6 mM FFA), and three drug intervention groups were established. The intervention groups were treated with 0.6 mM FFA in combination with either 50 μg/mL AS, 0.1 mM aminoguanidine hydrochloride (A) + 50 μg/mL AS, or 0.3 µM ruxolitinib (R) + 50 μg/mL AS. A and R are inhibitors of the AGE–RAGE and JAK1–STAT3 signaling pathways, respectively. Cells were seeded in triplicate and incubated in 1 mL of the respective medium at 37 °C with 5% CO_2_ for 24 h.

### 2.13 Oil red O staining

HepG2 cells were seeded in 24-well plates (6 × 10^4^ cells/plate, 1 mL/plate) and incubated for 24 h. After treatment as described in Section 2.1.2 for 24 h, the medium was removed, and cells were washed twice with PBS. Cells were fixed with Oil red O for 30 min, washed twice with ddH_2_O, and rinsed with 60% isopropanol for 20 s–30 s. Fresh Oil red O working solution was added and incubated for 20 min. The stain was removed, and cells were differentiated with 60% isopropanol for 30 s until background clearance, followed by five washes with ddH_2_O. The nuclei were counterstained with Mayer’s hematoxylin for 2 min, washed five times with ddH_2_O, and incubated with Oil red O buffer for 1 min. The buffer was replaced with ddH_2_O, and the stained cells were visualized under an inverted microscope.

### 2.14 Western blotting

Protein samples from both liver tissues (n = 3 per group) and HepG2 cells (n = 3 per group) were extracted separately using RIPA lysis buffer and maintained on ice. Equal quantities of these proteins were subjected to electrophoresis before being transferred to PVDF membranes. After being transferred, the membranes were immersed in blocking buffer (5% skim milk) for 2 h, followed by overnight incubation at 4 °C with the antibodies at a dilution of 1:1,000 for AGE, RAGE, ALOX15, ACSL4, GPX4, SLC7A11, FTH1, Nrf2, HO-1, JAK1, p-JAK1, STAT3, p-STAT3, and GAPDH at 4 °C. The following day, after washing the membranes, they were probed with the corresponding secondary antibody at a 1:3,000 dilution for 2 h. After further washing, images were obtained through enhanced chemiluminescence. The protein bands were detected using an enhanced chemiluminescence (ECL) detection system (reagent: BioSharp, BL523A; imager: Bio-Rad ChemiDoc MP). To ensure quantitative accuracy, multiple exposure time points were used to confirm that the chemiluminescent signals were within the linear range of detection for all proteins of interest. The absorbance values from the detection results were analyzed using ImageJ 1.53a software.

### 2.15 Molecular docking

In the preliminary stage of this research, the contents of isochlorogenic acid A, isochlorogenic acid B, and isochlorogenic acid C in AS were determined by HPLC. The content ranges of these three metabolites were found to be between 3.58% and 7.77%, and they were identified as the main metabolites in AS (Li et al., 2017; Maierdan et al., 2017). The chemical structures of isochlorogenic acid A, isochlorogenic acid B, and isochlorogenic acid C were obtained from the PubChem database (https://pubchem.ncbi.nlm.nih.gov/). The RAGE, JAK1, and STAT3 protein structures were downloaded from PDB (https://www.rcsb.org/). Molecular docking of chemical structure with protein structure was conducted using AutoDock Vina (Scripps Research Institute, La Jolla, United States), and the results were analyzed with AutoDock Tools (Scripps Research Institute, La Jolla, United States).

### 2.16 Statistical analysis

Statistical analysis was carried out using GraphPad Prism 6.0 software. The experimental results are presented as the mean ± standard deviation. One-way analysis of variance (ANOVA) was used for the analysis of differences among multiple groups, and Tukey’s test was employed to assess the differences between experimental groups. A minimum of three independent biological replicates were performed, with a sample size of n ≥ 3 per group. A *p*-value below 0.05 was regarded as statistically significant.

## 3 Result

### 3.1 AS alleviates liver injury and lipid metabolism disorder in MASH mice model

**FIGURE 1:**
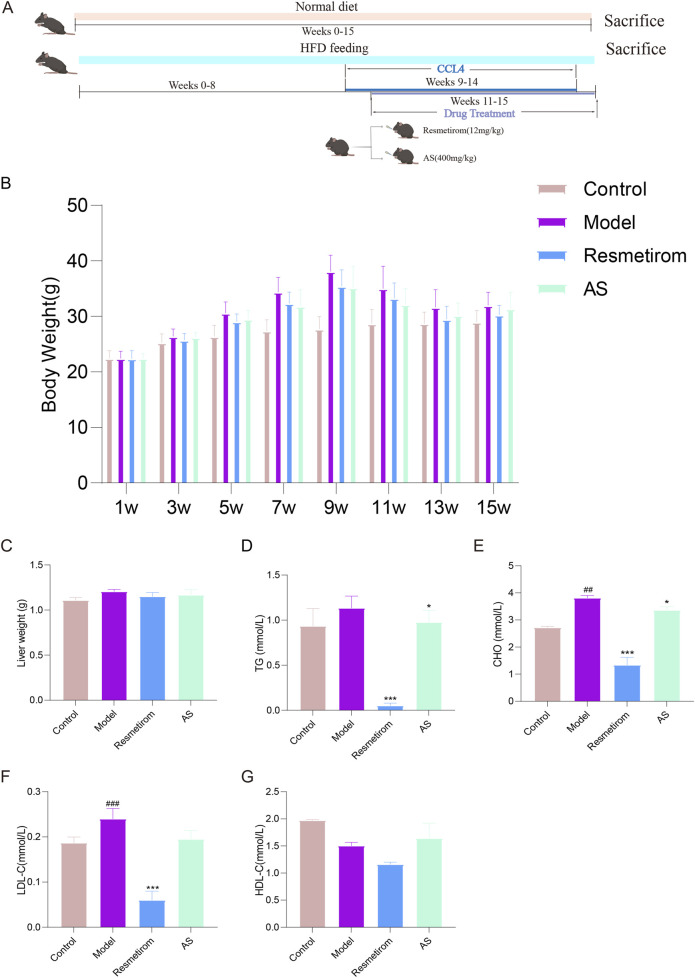
Effects of the dietary and pharmacological interventions on the body weight, liver weight, and blood lipid profiles in the MASH mice model. **(A)** Schematic diagram for inducing MASH in the mice model. **(B)** Body weight. **(C)** Liver weight of mice. **(D)** Concentrations of TG. **(E)** Concentrations of CHO. **(F)** Concentrations of LDL-C. **(G)** Concentrations of HDL-C in mice serum. ^##^ *P* < 0.01 and ^###^ *P* < 0.001 vs. control group; ^*^ *P* < 0.05, and ^***^ *P* < 0.001 vs. model group.

To evaluate the effects of AS against MASH, we used 40 mice on a HFD. From the ninth week, 1% CCL_4_ olive oil solution was used to induce MASH in the model. Drug administration was initiated in the 11th week, with mice receiving resmetirom (12 mg/kg) and AS (400 mg/kg) for a total treatment duration of 5 weeks (Figure 1A). We first explored the effect of drugs on the MASH mice model. As expected, the mice began to lose weight starting at 1 week after CCL_4_ induction (Figure 1B). This weight loss was an objective consequence of CCl_4_ induction, including reduced appetite, liver injury, and metabolic dysfunction. Notably, there were no significant differences in the body weight between the model group and the treatment groups. In addition, compared with that of the control group, the liver weight of mice with HFD/CCL_4_ was increased, and it decreased after AS treatment (Figure 1C). TG, CHO, HDL-C, and LDL-C are involved in hepatic lipid deposition and are key drivers of MASH occurrence. Mice treated with HFD/CCL_4_ developed dyslipidemia, showing increases in TG, CHO, and LDL-C alongside a decrease in HDL-C. However, administration of AS effectively counteracted these changes (Figures 1D–G).

**FIGURE 2:**
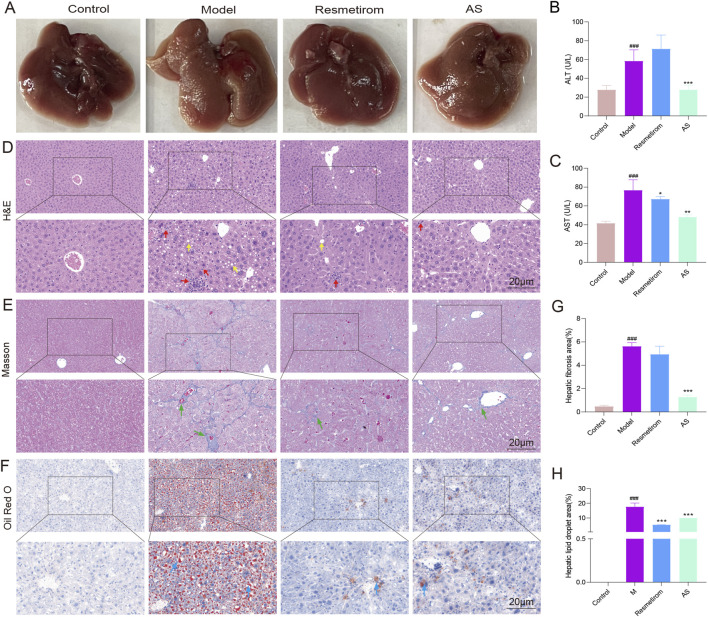
Evaluation of liver function serology and histopathology in the MASH mice model from different treatment groups. **(A)** Demonstration of liver lesions in the mice of each group. **(B)** Concentrations of ALT in mice serum. **(C)** Concentrations of AST in mice serum. **(D)** H&E staining of liver tissues. Cytoplasmic lipid vacuoles (yellow arrows) and focal inflammation (red arrows). **(E)** Masson staining of liver tissues and hepatic fibrosis (green arrows). **(F)** Oil red O staining of liver tissues and Oil red O-stained lipid droplets (blue arrows). **(G)** Hepatic fibrosis area percentage. **(H)** Hepatic lipid droplet area percentage. ^###^ *P* < 0.001 vs. control group; ^*^ *P* < 0.05, ^**^ *P* < 0.01, and ^***^ *P* < 0.001 vs. model group.

Meanwhile, the pale-brown coloration in the liver observed in the model mice was notably reversed by treatment with AS (Figure 2A). ALT and AST are considered the gold standards for liver injury. Treatment with AS reduced the elevated serum levels of ALT and AST in HFD/CCL_4_ mice (Figures 2B,C). In addition, histological analysis confirmed ballooning degeneration, inflammatory infiltration, hepatic fibrosis, and excessive lipid droplets in the livers of the MASH mice model (Figures 2D–F; indicated by yellow, red, green, and blue arrows, respectively). It is worth noting that AS mitigated the cytoplasmic lipid vacuoles and focal inflammatory infiltration and alleviated both hepatic fibrosis and Oil red O-positive lipid droplets (Figures 2G,H). Taken together, these results indicate that AS can effectively attenuate liver injury and lipid metabolism disorder in the MASH mice model.

### 3.2 AS ameliorates MASH by reversing hepatic metabolic perturbations

**FIGURE 3:**
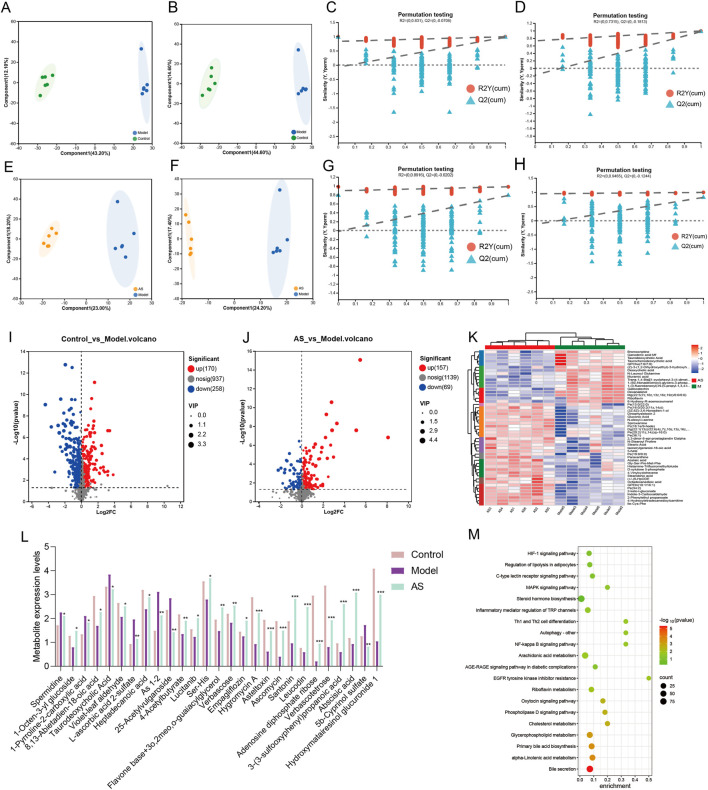
Metabolomics analysis reveals the therapeutic effects of AS on MASH. **(A,B)** OPLS-DA models: control vs. model [**(A)** ESI^+^; **(B)** ESI^−^]. **(C,D)** OPLS-DA permutation validation: control vs. models (**(C)**: ESI^+^; **(D)** ESI^−^), **(E,F)**. OPLS-DA model: model vs. AS [**(E)** ESI^+^; **(F)** ESI^−^]. **(G,H)** OPLS-DA permutation validation [**(G)** ESI^+^; **(H)** ESI^−^]. **(I,J)** Volcano plots of metabolites (FDR <0.05), highlighting the upregulated (red) and downregulated (blue) metabolites. **(K)** Heatmap analysis of differential metabolites (columns: groups; rows: metabolites; scale: Z-score). **(L)** Metabolite expression levels. **(M)** KEGG pathway enrichment analysis of differential metabolites between the model and the AS groups.

Metabolomics analysis was performed to characterize the associated metabolic alterations in the liver tissues of the MASH mice model after treatment with AS. The OPLS-DA models effectively differentiated the model group from the control and AS conditions in both the positive and negative ion modes (Figures 3A–H), indicating significant changes in metabolite levels. Subsequently, volcano plots visualized the metabolite changes in the liver, identifying 428 dysregulated metabolites between the normal and the model groups (170 upregulated and 258 downregulated) (Figure 3I) and 226 dysregulated metabolites between the AS and the model groups (157 upregulated and 69 downregulated) (Figure 3J). Heatmaps were generated to display the differentially abundant metabolites between the model and the AS groups (Figure 3K). Interestingly, among the 60 characteristic metabolites (VIP >1.0, *p* < 0.05) dysregulated between the control and model groups, 85% (37 upregulated and 14 downregulated) were normalized by treatment with AS (Figure 3L) (Table 1). As shown in Table 1, AS treatment normalized a panel of dysregulated metabolites in MASH mice. These metabolites encompass diverse origins, including endogenous metabolites involved in lipid and bile acid metabolism (e.g., taurodeoxycholic acid, heptadecanoic acid, and various phospholipids), plant-derived metabolites likely absorbed from AS (e.g., artabsin, 8,13-abietadien-18-oic acid, and ligustroflavone), and microbial-derived metabolites reflecting gut microbiota activity (e.g., muramic acid). The presence of plant-specific metabolites in the liver samples indicates systemic absorption of AS constituents, which may contribute to its pharmacological effects. Notably, the relative abundance of oxidized glutathione (GSSG) was downregulated following AS treatment (Supplementary Figure S2). It is well established that during ferroptosis, GPX4 consumes reduced glutathione (GSH), which reduces lipid peroxides, concomitantly generating GSSG. This result indicates that AS may suppress ferroptosis-related metabolic alterations.

**TABLE 1:** Characteristic metabolites dysregulated in the model and normalized by AS treatment.

​	Retention time	Metabolite	Formula	Origin	M/Z	Adducts	Model vs. Control			As vs. Model		
							VIP_pred_OPLS-DA	FC(M/C)	Trend	VIP_pred_OPLS-DA	FC(BHS/M)	Regulate
1	8.66695	As 1-2	C39H73NO9	A, M	744.5217509	M + FA-H	2.23	2.09	↑***	2.33	0.68	↓**
2	1.34425	L-ascorbic acid 2-sulfate	C6H8O9S	A	254.9793623	M-H	1.83	2.10	↑***	2.37	0.58	↓***
3	7.799516667	Ps(22:2 (13z, 16z)/20:1 (11z))	C48H88NO10P	A	892.5991198	M + Na	1.67	1.93	↑**	3.06	0.38	↓***
4	1.17025	Muramic acid	C9H17NO7	M	310.1141632	M + Hac-H	1.62	1.36	↑***	1.24	0.92	↓**
5	1.124933333	1-Pyrroline-2-carboxylic acid	C5H7NO2	A	114.054027	M + H	1.49	1.57	↑***	1.11	0.87	↓*
6	7.11805	Pc(18:3 (9,11,15)-oh (13)/22:4 (7z, 10z, 13z, 16z))	C48H82NO9P	A	906.5874999	M + Hac-H	1.45	1.84	↑*	3.00	0.34	↓***
7	9.01075	N-oleoyl-l-serine	C21H39NO4	A	368.2811856	M-H	1.37	1.28	↑**	1.73	1.18	↑**
8	7.510366667	15-Methylprostaglandin e1	C21H36O5	A	333.2418927	M + H-2H_2_O	1.27	0.77	↓**	1.65	1.26	↑**
9	5.041183333	Chinenoside V	C45H72O19	P	881.4452492	M + H-2H_2_O	1.21	1.52	↑**	1.25	1.19	↑*
10	7.8215	Lithocholenic acid	C24H38O3	A	375.2889257	M + H	1.16	1.21	↑**	1.45	0.87	↓**
11	6.234766667	5b-Cyprinol sulfate	C27H48O8S	A	567.279393	M + Cl	1.08	1.49	↑*	2.99	0.26	↓***
12	7.125383333	Pangamic acid	C20H40N2O8	P	435.2742528	M-H	1.01	1.16	↑**	2.04	0.76	↓**
13	1.148433333	Castavinol	C26H30O13	P	549.1667701	M-H	3.10	0.31	↓***	2.88	2.14	↑*
14	1.460233333	Verbascotetrose	C24H42O21	P	711.2203317	M + FA-H	3.08	0.14	↓***	2.48	3.40	↑*
15	1.46525	Fleroxacin N-oxide	C17H18F3N3O4	—	418.1550448	M + CH_3_OH + H	2.93	0.23	↓***	2.80	2.83	↑*
16	1.46525	Hygromycin A	C23H29NO12	M	476.1600068	M + H-2H_2_O	2.82	0.18	↓***	2.93	3.91	↑*
17	1.19925	Virginiamycin m1	C28H35N3O7	M	560.2198681	M + Cl	2.69	0.36	↓***	2.53	1.95	↑*
18	1.429083333	Ligustroflavone	C33H40O18	P	689.211833	M + H-2H_2_O	2.68	0.26	↓***	2.45	2.42	↑*
19	1.117433333	Melezitose	C18H32O16	P	505.1757967	M + H	2.51	0.36	↓***	1.81	1.53	↑*
20	1.117433333	NeuAc (alpha->6)GalNAc(alpha1- > O)Ser	C22H37N3O16	A	654.2286924	M + CH_3_OH + Na	2.46	0.43	↓***	2.18	1.57	↑**
21	1.429083333	Hydroxyrubicin	C27H28O12	—	545.167273	M + H	2.43	0.46	↓***	2.37	1.65	↑*
22	1.1101	Lucitanib	C26H25N3O4	—	448.1657442	M + Na-H_2_O	2.28	0.45	↓***	2.12	1.66	↑*
23	1.139433333	Verbascose	C30H52O26	P	867.2382625	M + K	2.21	0.56	↓***	2.06	1.46	↑*
24	1.153933333	Empagliflozin	C23H27ClO7	—	455.1210962	M + Na-H_2_O	2.20	0.40	↓**	2.23	1.94	↑*
25	3.721266667	Gentiopicroside	C16H20O9	P	377.0872376	M + Na-2H	2.07	0.25	↓***	1.44	1.97	↑*
26	1.712066667	Reduced haloperidol	C21H25ClFNO2	A	360.1497115	M + H-H_2_O	2.00	0.65	↓***	1.68	1.24	↑*
27	1.690383333	6-Chloro-2′,3′-dideoxyguanosine	C10H14ClN5O3	A	619.1496583	2M + FA-H	1.99	0.60	↓***	1.55	1.24	↑*
28	1.6614	Epiafzelechin-(4alpha->8)-pelargonidin 3′-glucoside	C36H33O15+	P	726.1534017	M + Na-2H	1.98	0.54	↓***	1.50	1.31	↑*
29	6.8904	Ulimorelin	C30H39FN4O4	—	602.3109606	M + ACN + Na	1.86	0.49	↓***	1.97	1.65	↑*
30	7.58435	1-Hydroxyvitamin D5	C29H48O2	—	411.3584225	M + H-H_2_O	1.86	2.00	↑***	1.66	1.24	↑**
31	6.7084	Dinophysistoxin 2	C44H68O13	A	787.4547072	M + H-H_2_O	1.82	0.69	↓***	2.21	1.33	↑**
32	1.082766667	Met-Tyr	C14H20N2O4S	A	311.1087511	M-H	1.75	0.54	↓***	1.12	0.78	↓*
33	10.72765	Cer(t18:0/18:1 (12Z)-O (9S,10R))	C36H69NO5	A	560.5010527	M + H-2H_2_O	1.71	1.82	↑**	1.22	1.14	↑**
34	1.141266667	Paraxanthine	C7H8N4O2	A, M	161.0452785	M-H_2_O-H	1.67	0.77	↓***	1.35	1.12	↑*
35	7.51685	Pc(22:4 (7z,10z,13z,16z)/18:3 (10,12,15)-oh (9))	C48H82NO9P	A	906.5791142	M + Hac-H	1.65	1.62	↑*	2.76	0.60	↓**
36	1.141266667	Cyclo (Ac-cys-asn-dmt-amf-gly-asp-cys)	C34H47N9O12S3	—	850.2272072	M-H_2_O-H	1.64	0.54	↓***	1.78	1.53	↑*
37	9.830533333	Heptadecanoic acid	C17H34O2	A	269.2489586	M-H	1.58	0.75	↓***	1.80	1.21	↑***
38	7.654333333	7-Ketolithocholic acid	C24H38O4	A	435.273081	M + FA-H	1.54	1.42	↑***	1.33	0.86	↓*
39	5.628816667	4-Acetylbutyrate	C6H10O3	A	259.1190569	2M-H	1.51	0.62	↓***	1.79	1.41	↑**
40	6.723233333	Oligomycin	C45H74O11	A	813.5132862	M + Na	1.43	0.74	↓**	1.66	1.25	↑**
41	5.642983333	Piliformic acid	C11H18O4	A	215.127435	M + H	1.37	0.69	↓***	2.03	1.41	↑***
42	5.5853	Octopine	C9H18N4O4	A	281.100521	M + Cl	1.37	0.78	↓***	1.80	1.22	↑***
43	5.584983333	Violet-leaf aldehyde	C9H14O	P	121.1002268	M + H-H_2_O	1.33	0.78	↓***	1.73	1.21	↑***
44	7.132533333	17-Estradiol cyclooctyl acetate	C28H40O3	A	459.2706805	M + Cl	1.29	1.24	↑***	2.68	0.65	↓***
45	10.78548333	GPEtn (18:1/16:1)	C39H74NO8P	A	716.525124	M + H	1.21	0.83	↓***	1.85	1.22	↑***
46	3.1113	6-Hydroxy flavin adenine dinucleotide	C27H33N9O16P2	A	800.1454693	M-H	1.16	1.37	↑**	2.34	1.38	↑***
47	5.055683333	2,2'-[Cyclohexane-1,4-diylbis (methanediyloxymethanediyl)]dioxirane	C14H24O4	A	274.2011069	M + NH_4_	1.13	0.87	↓***	1.70	1.15	↑***
48	0.962616667	1-Octen-3-yl glucoside	C14H26O6	P	295.1497969	M + Na-H_2_O	1.09	0.62	↓**	2.08	1.86	↑**
49	3.1258	Secologanate	C16H22O10	P	419.1201065	M + FA-H	1.06	1.35	↑**	1.97	1.29	↑***
50	8.109483333	5-hete	C20H32O3	A	365.2330364	M + FA-H	2.10	0.60	↓**	2.07	1.39	↑*
51	7.317866667	8,13-Abietadien-18-oic acid	C20H30O2	P	303.2315376	M + H	2.00	0.58	↓***	1.90	1.34	↑**
52	2.977983333	Methacycline	C22H22N2O8	A	460.1747238	M + NH_4_	1.80	0.35	↓**	2.57	2.75	↑**
53	5.3095	Thr-Ile-Asp-Phe-Glu	C28H41N5O11	A	624.2949353	M + H	1.68	0.68	↓***	1.70	1.30	↑*
54	6.12895	Artabsin	C15H20O3	P	249.1483064	M + H	1.61	0.51	↓**	3.10	2.44	↑***
55	4.889016667	Indole-3-acetaldehyde	C10H9NO	P	160.0746449	M + H	1.58	1.71	↑**	2.16	1.27	↑***
56	5.933783333	Histamine-trifluoromethyltoluide	C19H25F3N4O	A	427.1963722	M + FA-H	1.46	0.79	↓***	1.53	1.16	↑**
57	3.548966667	L-DOPA 3′-glucoside	C15H21NO9	P	423.1364374	M + ACN + Na	1.18	1.55	↑**	1.53	1.30	↑*
58	7.415366667	25-Acetylvulgaroside	C27H42O7	P	459.2707431	M-H_2_O-H	1.12	1.21	↑**	3.14	0.50	↓***
59	0.97745	Piperacillin	C23H27N5O7S	A	559.19844	M + ACN + H	1.03	0.64	↓*	1.50	1.56	↑*
60	6.591583333	Taurodeoxycholic acid	C26H45NO6S	A	500.3026928	M + H	1.01	1.15	↑*	1.78	0.84	↓**

P, plant origin; A, animal origin; M, microbial origin, /, synthetic. *p < 0.05, **0.01 < p < 0.05, and ***p < 0.01.

To further elucidate the changes in metabolic biomarker levels in the HFD-/CCL_4_-induced mice after AS intervention, we conducted metabolic pathway enrichment analysis on significantly altered liver metabolites. The enrichment results revealed significant involvement of the following pathways: bile secretion, alpha-linolenic acid metabolism, primary bile acid biosynthesis, glycerophospholipid metabolism, phospholipase D signaling pathway, arachidonic acid metabolism, NF-kappa B signaling pathway, AGE–RAGE signaling pathway, and inflammatory mediator regulation of TRP channels (IMR–TRP) (Figure 3M).

Taken together, these results indicate that AS may improve MASH by reversing the differential metabolites in the MASH mice model and regulating the lipid metabolism pathways and inflammatory pathways enriched with these differential metabolites.

### 3.3 Transcriptomics reveals the key biological processes and pathways of AS in the MASH mice model

**FIGURE 4:**
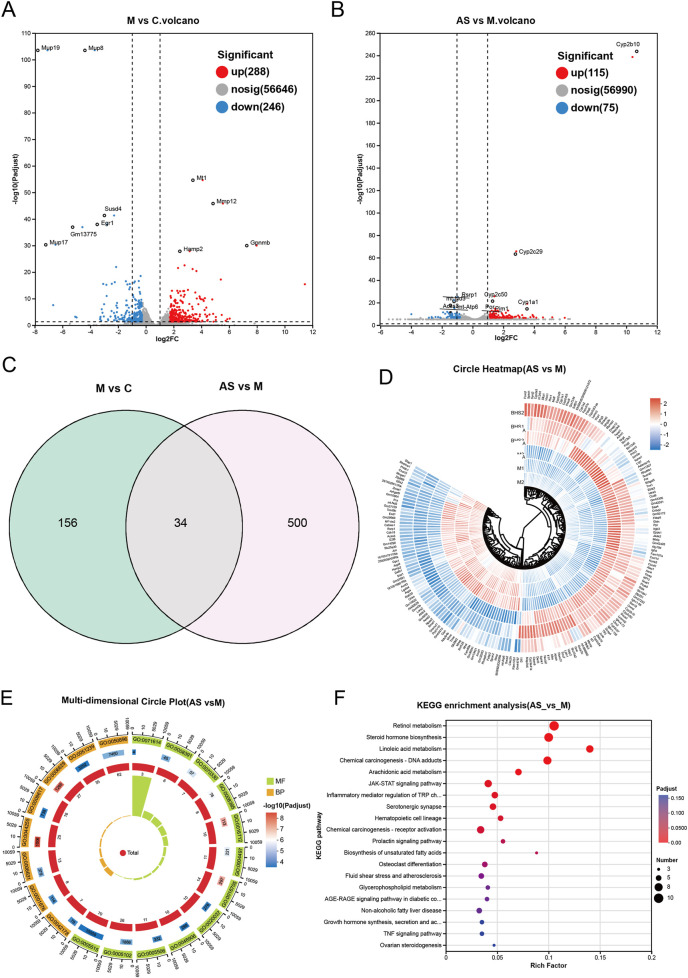
Transcriptomics reveals key biological processes and pathways of AS in intervening against MASH. **(A,B)** mRNA volcano plots, highlighting the upregulated (red) and downregulated (blue) mRNAs. **(C)** Venn diagram analysis. **(D)** Circle heatmaps of DEGs between the model group and the AS group. **(E)** GO enrichment analysis. **(F)** KEGG pathway enrichment analysis.

RNA-seq was used to further clarify the potential mechanisms underlying the therapeutic effects of AS on MASH. Volcano plots showed that there were 288 upregulated and 246 downregulated DEGs between the control and the model groups and 115 upregulated and 75 downregulated DEGs between the model and the AS groups (Figures 4A,B). There were 30 intersecting genes between the control group vs. model group and the model group vs. AS group (Figure 4C; Table 2). Clustering heatmaps visually displayed the differential levels of gene expression between the model and AS groups (Figure 4D). To explore the potential functions of the DEGs, GO enrichment analysis was performed. GO terms with a FDR <0.05 and a gene count ≥5 were considered significantly enriched. The enrichment results for biological processes (BPs), cellular components (CCs), and molecular functions (MFs) are shown in Figure 4E. Notably, DEGs showed primary enrichment in MFs, such as catalytic activity and oxidoreductase activity; BPs, particularly those involved in lipid metabolism, fatty acid metabolic, and lipid biosynthetic processes; and CCs, such as cellular anatomical entity, symbiont-containing vacuole membrane, and signal recognition particle (Figure 4E).

**TABLE 2:** Characteristic genes dysregulated in the model and normalized by AS treatment.

​	Gene ID	Gene name	Gene description	M_vs._C	AS_vs._M
1	ENSMUSG00000009092	Derl3	Der1-like domain family, member 3 [source: MGI symbol; Acc: MGI:1917627]	↓	↑
2	ENSMUSG00000020571	Pdia6	Protein disulfide isomerase associated 6 [source: MGI symbol; Acc: MGI:1919103]	↓	↑
3	ENSMUSG00000044254	Pcsk9	Proprotein convertase subtilisin/kexin type 9 [source: MGI symbol; Acc: MGI:2140260]	↓	↑
4	ENSMUSG00000022351	Sqle	Squalene epoxidase [source: MGI symbol; Acc: MGI:109296]	↓	↑
5	ENSMUSG00000024029	Tff3	Trefoil factor 3, intestinal [source: MGI symbol; Acc: MGI:104638]	↓	↑
6	ENSMUSG00000022769	Sdf2l1	Stromal cell-derived factor 2-like 1 [source: MGI symbol; Acc: MGI:2149842]	↓	↑
7	ENSMUSG00000095079	Igha	Immunoglobulin heavy constant alpha [source: MGI symbol; Acc: MGI:96444]	↓	↑
8	ENSMUSG00000025002	Cyp2c55	Cytochrome P450, family 2, subfamily c, polypeptide 55 [source: MGI symbol; Acc: MGI:1919332]	↓	↑
9	ENSMUSG00000042246	Tmc7	Transmembrane channel-like gene family 7 [source: MGI symbol; Acc: MGI:2443317]	↓	↑
10	ENSMUSG00000038599	Capn8	Calpain 8 [source: MGI symbol; Acc: MGI:2181366]	↓	↑
11	ENSMUSG00000042248	Cyp2c37	Cytochrome P450, family 2. subfamily c, polypeptide 37 [source: MGI symbol; Acc: MGI:1306806]	↓	↑
12	ENSMUSG00000042638	Gucy2c	Guanylate cyclase 2c [Source: MGI symbol; Acc: MGI:106903]	↑	↑
13	ENSMUSG00000018459	Slc13a3	Solute carrier family 13 (sodium-dependent dicarboxylate transporter), member 3 [source: MGI symbol; Acc: MGI:2149635]	↑	↑
14	ENSMUSG00000032315	Cyp1a1	Cytochrome P450, family 1, subfamily a, polypeptide 1 [source: MGI symbol; Acc: MGI:88588]	↓	↑
15	ENSMUSG00000076613	Ighg2b	Immunoglobulin heavy constant gamma 2B [source: MGI symbol; Acc: MGI: 96445]	↓	↑
16	ENSMUSG00000022025	Cnmd	Chondromodulin [source: MGI symbol; Acc: MGI:1341171]	↓	↑
17	ENSMUSG00000052837	Junb	Jun B proto-oncogene [source: MGI symbol; Acc: MGI:96647]	↓	↑
18	ENSMUSG00000085834	Gm15622	Predicted gene 15622 [source: MGI symbol; Acc: MGI:3783067]	↑	↓
19	ENSMUSG00000056978	Hamp2	Hepcidin antimicrobial peptide 2 [source: MGI symbol; Acc: MGI:2153530]	↑	↓
20	ENSMUSG00000029254	Stap1	Signal-transducing adapter family member 1 [source: MGI symbol; Acc: MGI:1926193]	↑	↓
21	ENSMUSG00000027399	Il1a	Interleukin 1 alpha [source: MGI symbol; Acc: MGI:96542]	↑	↓
22	ENSMUSG00000037411	Serpine1	Serine (or cysteine) peptidase inhibitor, clade E, member 1 [source: MGI symbol; Acc: MGI:97608]	↑	↓
23	ENSMUSG00000040420	Cdh18	Cadherin 18 [source: MGI symbol; Acc: MGI:1344366]	↑	↓
24	ENSMUSG00000108822	Gm44787	Predicted gene 44787 [source: MGI symbol; Acc: MGI:5753363]	↑	↓
25	ENSMUSG00000034220	Gpc1	Glypican 1 [source: MGI symbol; Acc: MGI:1194891]	↑	↓
26	ENSMUSG00000037071	Scd1	Stearoyl-coenzyme a desaturase 1 [source: MGI symbol; Acc: MGI:98239]	↑	↓
27	ENSMUSG00000032578	Cish	Cytokine-inducible SH2-containing protein [source: MGI symbol; Acc: MGI:103159]	↑	↓
28	ENSMUSG00000039220	Ppp1r10	Protein phosphatase 1, regulatory subunit 10 [source: MGI symbol; Acc: MGI:1289273]	↑	↓
29	ENSMUSG00000044748	Defb1	Defensin beta 1 [source: MGI symbol; Acc: MGI:1096878]	↑	↓
30	ENSMUSG00000040957	Cables1	CDK5 and abl enzyme substrate 1 [source: MGI symbol; Acc: MGI:1927065]	↑	↓

↑indicates upregulated; ↓indicates downregulated.

The KEGG enrichment results indicated that the DEGs were predominantly enriched in pathways related to lipid metabolism and inflammatory responses. These included metabolic processes such as linoleic acid metabolism, arachidonic acid metabolism, unsaturated fatty acid biosynthesis, and glycerophospholipid metabolism. Significant enrichment was also observed in key signaling pathways, including AGE–RAGE, JAK–STAT, IMR–TRP, and TNF signaling (Figure 4F).

KEGG analysis revealed that the DEGs were primarily associated with lipid metabolism and inflammatory pathways, including arachidonic acid metabolism, linoleic acid metabolism, biosynthesis of unsaturated fatty acids, glycerophospholipid metabolism, and signaling pathways such as AGE–RAGE, JAK–STAT, IMR–TRP, and TNF (Figure 4F). These results indicate that AS alleviates MASH by regulating lipid metabolism and inflammation at the mRNA level.

### 3.4 Integrated analysis of metabolomics and transcriptomics reveals the therapeutic effects of AS on MASH

**FIGURE 5:**
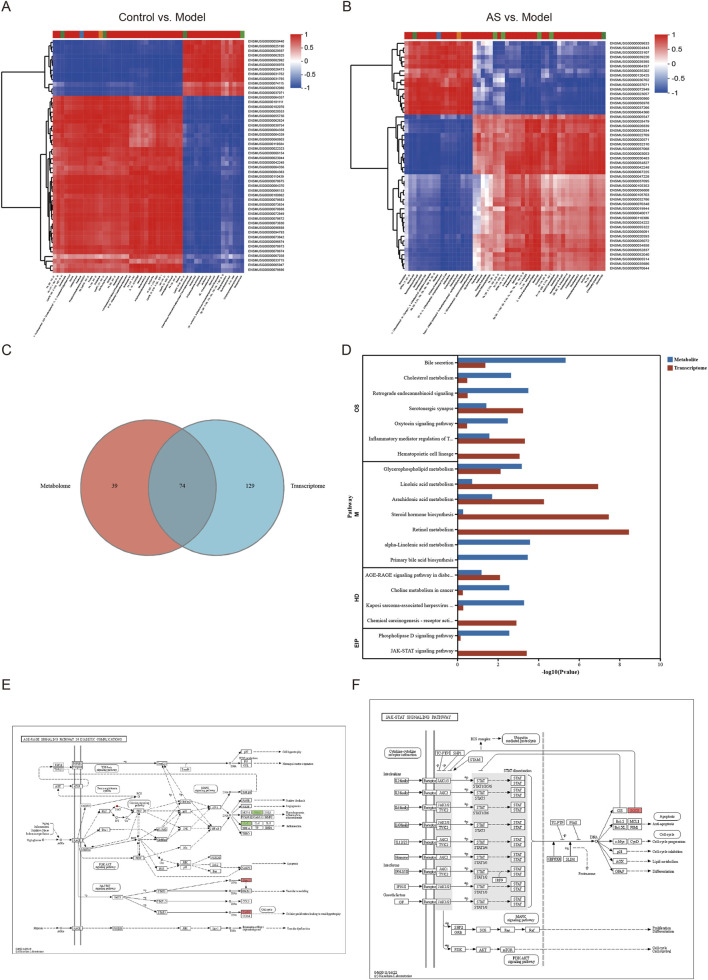
Combined analysis of metabolomics and transcriptomics. **(A,B)** Heatmaps showing the combined analysis results of DEGs and differential metabolites [**(A)** control and model; **(B)** model and AS group; red indicates a positive correlation, while blue indicates a negative correlation]. **(C)** Venn diagram analysis. **(D)** KEGG pathway enrichment analysis of differential metabolites between the model and the AS groups. **(E,F)** AGE–RAGE and JAK–STAT pathways, highlighting upregulated (red) and downregulated (green) ones.

We integrated the hepatic transcriptomic and metabolomic data via KEGG mapping to identify metabolite–gene pairs with significant correlations (*p* < 0.05). Pearson’s correlation analysis was performed to assess the linear relationships between DEGs and differentially abundant metabolites. Correlation pairs with |r| ≥ 0.7 and *p* < 0.05 were considered statistically significant and were retained for subsequent network construction. Comparative analyses were performed between the control and model groups and between the model and AS groups, with the differential metabolites plotted on the horizontal axis and the differential genes plotted on the vertical axis. Between the control group (C) and the model group (M), 12DEGs were positively correlated with 16 differential abundance metabolites and negatively correlated with 34 differential metabolites; simultaneously, 38 DEGs were positively correlated with 34 differential abundance metabolites and negatively correlated with 16 differential abundance metabolites (Figure 5A). Between the model group and the AS group, 16 DEGs were positively correlated with 17 differential abundance metabolites and negatively correlated with 33 differential abundance metabolites; meanwhile, 34 DEGs were positively correlated with 33 differential abundance metabolites and negatively correlated with 17 differential abundance metabolites (Figure 5B).

To explore the common pathways altered in different groups, enrichment analysis was performed to construct potential pathways. A total of 20 signaling pathways were constructed, including oxidative stress and inflammation-related pathways, namely, JAK–STAT and AGE–RAGE signaling pathways; lipid metabolism-related pathways, namely, cholesterol metabolism and glycerophospholipid metabolism; bile acid and gut–liver axis-related pathways, namely, primary bile acid biosynthesis and bile secretion; fibrosis pathways, namely, retinol metabolism; and hormone and systemic regulation pathways; and weakly associated pathways such as chemical carcinogenesis, indicating that these 20 pathways may be crucial for AS to counteract MASH. The AGE–RAGE pathway is a driver of lipid metabolism and oxidative stress (Fernando et al., 2019), and the JAK–STAT3 signaling pathway acts as a key regulator of lipid metabolism and oxidative stress (Zhang et al., 2025). Therefore, we focused our study on this pathway. The significantly altered metabolites following AS intervention include DG [22:1 (13Z)/22:6 (4Z, 7Z, 10Z, 13Z ,16Z, 19Z)/0:0] and DG [18:3 (9Z, 12Z, 15Z)/16:0/0:0]. Meanwhile, the DEGs associated with the AGE–RAGE pathway after AS intervention comprise Pim1, CycD1, IL-1α, and PAI-1, and those associated with the JAK–STAT pathway comprise receptor and SOCS.

### 3.5 AS inhibits oxidative stress and inflammation in the MASH mice model via the AGE–RAGE signaling pathway

**FIGURE 6:**
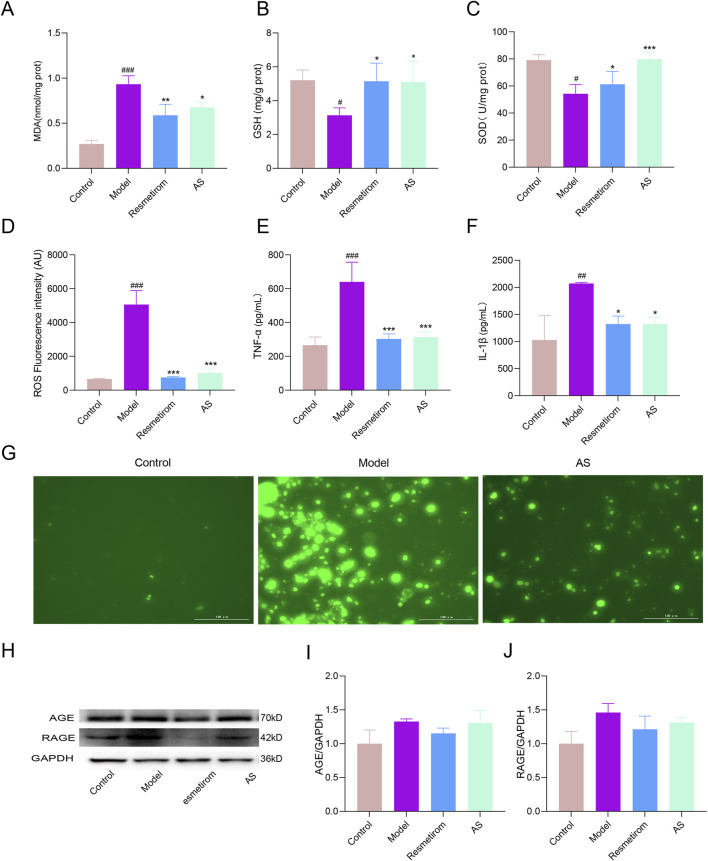
AS alleviates the disorder of lipid metabolism and inflammation in the MASH mice model. **(A–D)** Concentrations of MDA, GSH, SOD, and ROS in the liver tissues. **(E,F)** Concentrations of TNF-α and IL-1β in serum. **(G)** Fluorescence microscopy to detect the ROS level (green, ROS. Scale bar: 100 μm). **(H–J)** Western blotting detection of the expression levels of AGE and RAGE. ^#^ *P* < 0.05, ^##^ *P* < 0.01, and ^###^ *P* < 0.001 vs. control group; ^*^ *P* < 0.05, ^**^ *P* < 0.01, and ^***^ *P* < 0.001 vs. model group.

Inflammation and lipid dysregulation are well recognized as the core drivers of MASH progression (Carli et al., 2024). Oxidative stress, in particular, serves as a pivotal mechanism underlying liver injury as it not only induces hepatic lipid metabolic disorders but also amplifies inflammatory responses (Li et al., 2025). Consistent with these pathological features, we observed a significant accumulation of MDA—a classic biomarker of lipid peroxidation—in liver tissues from the HFD-/CCL_4_-induced MASH mice model (Figure 6A). Concurrently, the activity of key antioxidant enzymes, including GSH and SOD, was markedly reduced, accompanied by excessive ROS production (Figures 6B–D). These oxidative stress-related abnormalities were closely associated with elevated levels of pro-inflammatory cytokines, specifically TNF-α and IL-1β (Figures 6E,F), which was further validated by direct visualization of increased ROS fluorescence intensity via fluorescence microscopy (Figure 6G). Notably, AS intervention effectively normalized all these pathological alterations, restoring antioxidant capacity, suppressing ROS overproduction, and reducing pro-inflammatory cytokine levels. Collectively, these findings strongly support that AS exerts a protective effect against MASH by mitigating oxidative stress and alleviating inflammation.

To further elucidate the underlying molecular mechanisms, we analyzed the involvement of the AGE–RAGE pathway in AS-mediated protection against MASH. As shown in Figures 6H–J, hepatic levels of AGEs and their receptor (RAGE) were upregulated in HFD-/CCL_4_-induced mice, indicating robust activation of the AGE–RAGE pathway. In contrast, AS treatment substantially reduced the hepatic abundance of both AGEs and RAGE, indicating that inhibition of the AGE–RAGE pathway activation may contribute to the protective effects of AS in MASH.

### 3.6 AS attenuates MASH by inhibiting the AGE–RAGE signaling pathway, suppressing ferroptosis

**FIGURE 7:**
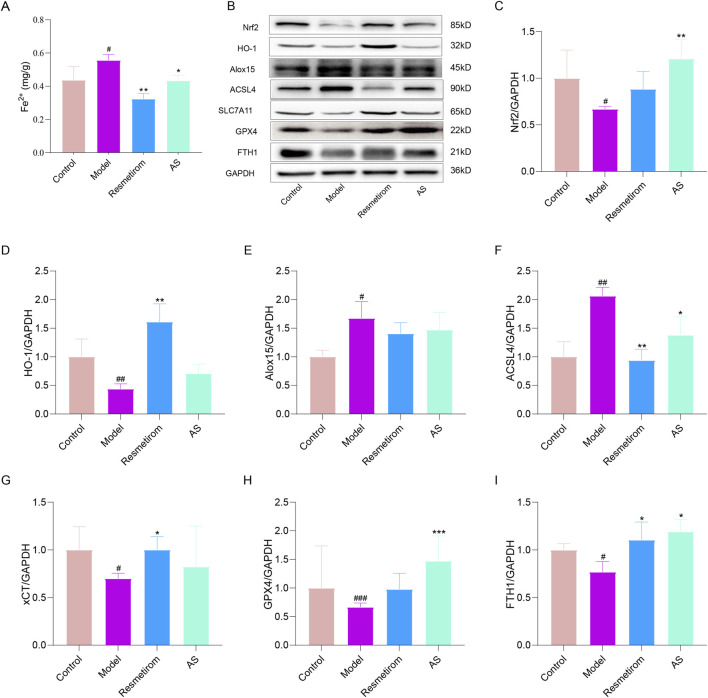
AS inhibits ferroptosis, thereby ameliorating MASH. **(A)** Fe^2+^ content in the liver tissue was measured using a commercial assay kit. **(B–I)** Western blotting detection of the expression levels of Nrf2, HO-1, SLC7A11, GPX4, FTH1, and GAPDH in the liver tissues. ^#^ *P* < 0.05, ^##^ *P* < 0.01, and ^###^ *P* < 0.001 vs. control group; ^*^ *P* < 0.05, ^**^ *P* < 0.01, and ^***^ *P* < 0.001 vs. model group.

Accumulating evidence indicates that ferroptosis is closely implicated in chronic liver injury, fibrosis, and inflammation, and it serves as a critical pathogenic driver in the progression of MASH (Chen et al., 2022). More importantly, the activated AGE–RAGE signaling pathway can promote oxidative stress, thereby inducing ferroptosis. Based on this, we hypothesized that AS may exert a protective effect against MASH progression by targeting and inhibiting ferroptosis. As anticipated, in liver tissues from the HFD-/CCL_4_-induced MASH mice model, the expressions of Nrf2 and HO-1—key regulators of the antioxidant stress response—were significantly downregulated. Concurrently, AS treatment effectively reduced hepatic Fe^2+^ accumulation in the MASH mice model (Figure 7A), which is a hallmark of ferroptosis. Furthermore, the model group showed a marked upregulation of ALOX15 and ACSL4 expressions, two critical pro-ferroptotic proteins. More importantly, the expression levels of ferroptosis suppressors, including solute carrier family GPX4, SLC7A11, and FTH1, were significantly suppressed in HFD-/CCL_4_-induced mice. Notably, all these ferroptosis-related pathological alterations were substantially reversed following AS intervention (Figures 7B–I). Collectively, these results strongly indicate that AS alleviates MASH progression by inhibiting the AGE–RAGE signaling pathway, thereby suppressing ferroptosis in hepatocytes.

### 3.7 AS regulates the JAK1–STAT3 signaling pathway in the MASH mice model

**FIGURE 8:**
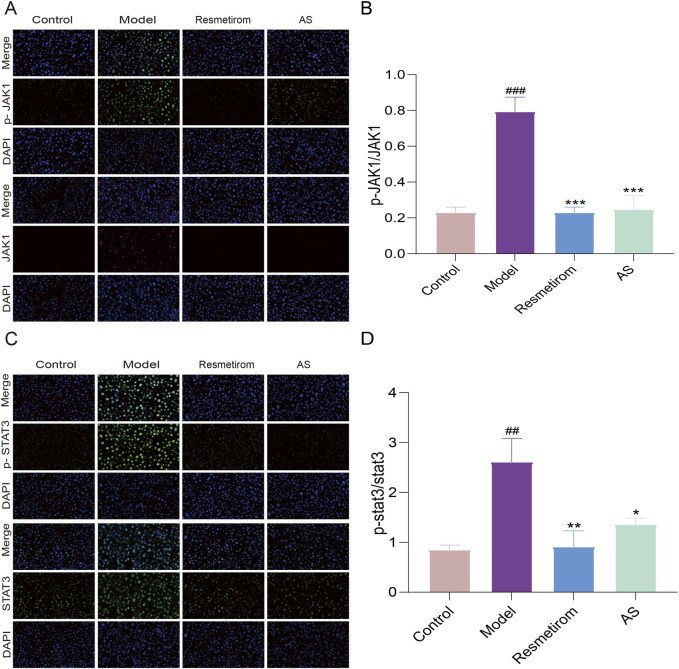
AS improves MASH via the JAK1–STAT3 signaling pathway. **(A,B)** Immunofluorescence detection of p-JAK1 and JAK1 expression levels in the liver tissues. **(C,D)** Immunofluorescence detection of p-STAT3 and STAT3 expression levels in the liver tissues. ^##^ *P* < 0.01 and ^###^ *P* < 0.001 vs. control group; ^*^ *P* < 0.05, ^**^ *P* < 0.01, and ^***^ *P* < 0.001 vs. model group.

The JAK–STAT3 pathway is closely associated with the pathogenesis of inflammation. Subsequently, we further verified whether AS is involved in the inhibition of MASH by regulating the JAK–STAT pathway. Our results demonstrated that the ratios of p-JAK1/JAK1 and p-STAT3/STAT3 showed a marked upregulation in mice administered HFD/CCL_4_, which indicated the activation of the JAK1–STAT3 signaling pathway. However, the JAK1–STAT3 signaling pathway was inhibited following treatment with AS (Figures 8A–D). These results indicate that AS alleviates inflammation by regulating the JAK1–STAT3 signaling pathway in MASH.

### 3.8 AS regulates the AGE–RAGE and JAK1–STAT3 signaling pathways *in vitro*

**FIGURE 9:**
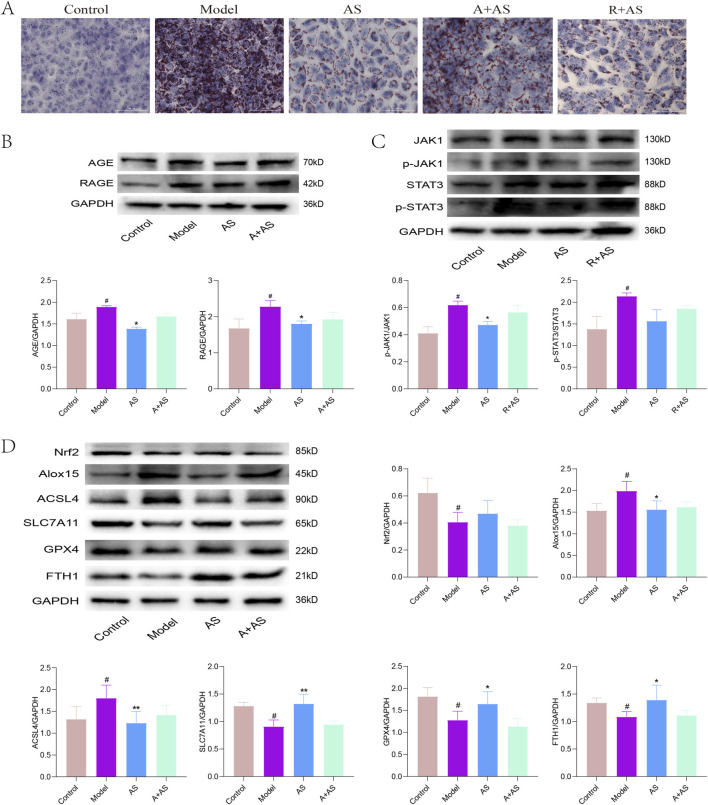
AS regulates the AGE–RAGE and JAK1–STAT3 signaling pathways *in vitro*. **(A)** Cell Oil Red O staining was performed to evaluate lipid droplet accumulation in FFA-treated HepG2 cells. **(B–D)** Western blotting was conducted to detect the protein levels of p-JAK1, JAK1, p-STAT3, STAT3, Nrf2, HO-1, SLC7A11, GPX4, FTH1, and GAPDH in HepG2 cells. ^#^ *P* < 0.05 vs. control group; ^*^ *P* < 0.05, ^**^ *P* < 0.01, and ^***^ *P* < 0.001 vs. model group.

To further elucidate the role of AS in the regulation of AGE–RAGE and JAK1–STAT3 signaling pathways in MASH *in vitro*, we established a MASH cellular model by stimulating HepG2 cells with FFA. The cells were then treated with AS, alone or in combination with an AGE–RAGE pathway inhibitor (A) or a JAK1–STAT3 pathway inhibitor (R). Oil red O staining results, shown in Figure 9A, revealed that FFA induction led to substantial intracellular lipid accumulation in HepG2 cells compared to the control group. AS treatment alone effectively suppressed FFA-induced lipid deposition; however, this effect was reversed upon the addition of either A or R. Furthermore, Western blotting analysis demonstrated that FFA stimulation upregulated the protein expressions of AGE and RAGE in HepG2 cells, while AS treatment downregulated their expression—a trend that was reversed by the addition of A (Figure 9B). Additionally, we observed that the ratios of p-JAK1/JAK1 and p-STAT3/STAT3 were increased in FFA-induced HepG2 cells, and these increases were attenuated by AS treatment. Notably, this attenuation was reversed by the addition of R (Figure 9C). Taken together, these findings indicate that AS ameliorates FFA-induced lipid deposition in HepG2 cells by regulating the AGE–RAGE and JAK1–STAT3 signaling pathways.

Furthermore, we explored the effect of AS on ferroptosis through the regulation of the AGE–RAGE pathway. Western blotting results, shown in Figure 9D, revealed that the protein levels of the antioxidant stress response regulator Nrf2 and the ferroptosis suppressors GPX4, SLC7A11, and FTH1 were decreased in FFA-induced HepG2 cells, while the protein levels of the pro-ferroptotic factors ALOX15 and ACSL4 were increased. However, AS treatment significantly upregulated the protein levels of Nrf2, GPX4, SLC7A11, and FTH1 and inhibited the protein expression levels of ALOX15 and ACSL4. Interestingly, these effects were reversed upon the addition of A. Taken together, these findings indicate that AS inhibits lipid deposition and suppresses ferroptosis by regulating the AGE–RAGE and JAK1–STAT3 signaling pathways *in vitro*.

### 3.9 The main metabolites of AS exhibit strong binding energy with the AGE–RAGE and JAK1–STAT3 signaling pathways

**FIGURE 10:**
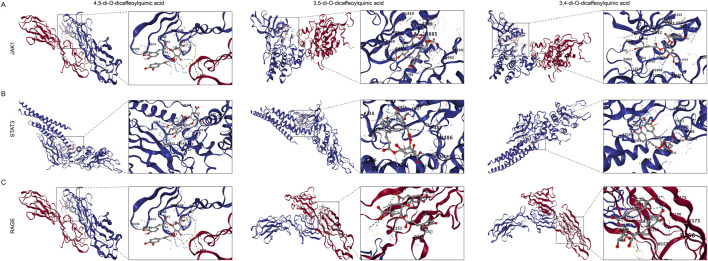
The main metabolites of AS exhibit strong binding energy with the AGE–RAGE and JAK1–STAT3 signaling pathways. **(A–C)** RAGE, JAK1, and STAT3, as key targets in the AGE–RAGE and JAK1–STAT3 signaling pathways, were subjected to molecular docking with the main chemical metabolites of AS, namely, isochlorogenic acid **(A)**, isochlorogenic acid **(B)**, and isochlorogenic acid **(C)**.

RAGE, JAK1, and STAT3, as key targets in the AGE–RAGE and JAK1–STAT3 signaling pathways, were subjected to molecular docking with the main chemical metabolites of AS, namely, isochlorogenic acid A, isochlorogenic acid B, and isochlorogenic acid C. A lower binding energy between the active metabolites and core targets indicates more stable binding. A docking binding energy of <−5 kcal mol^-1^ indicates a strong binding interaction. The results of our molecular docking study are shown in Figure 10. The binding energies between isochlorogenic acid A, isochlorogenic acid B, and isochlorogenic acid C and JAK1, STAT3, and RAGE were all <−5 kcal mol^-1^ (Figures 10A–C), indicating strong binding interactions. Notably, isochlorogenic acid C exhibited even stronger binding interactions with RAGE, JAK1, and STAT3, with binding energies of −6.7 kcal/mol, −9.2 kcal/mol, and −8.1 kcal/mol, respectively. These results indicate that AS’s main metabolites, namely, isochlorogenic acids A, B, and C, may have protective effects against MASH by regulating the JAK1–STAT3 and AGE–RAGE pathways.

## 4 Discussion

As a progressive metabolic dysfunction-associated steatotic liver disease, MASH exhibits a complex pathogenesis (Harrison et al., 2024a), involving multiple pathological processes such as lipid metabolism disorder, inflammatory response, and oxidative stress. Currently, clinical therapeutic options for MASH remain limited. Natural medicines, endowed with unique advantages of multicomponent composition, multitarget action, and low toxicity, have demonstrated broad application prospects in the treatment of liver diseases (Sun et al., 2022). *A*. *scoparia*, a traditional Chinese botanical drug, has long been applied in the management of hepatobiliary disorders. Contemporary pharmacological evidence supports its multiple bioactivities, such as antioxidant, anti-inflammatory, and hepatoprotective functions (Ding et al., 2021; Yoon et al., 2023; Wang et al., 2024). However, its therapeutic efficacy and underlying molecular mechanisms against MASH have not yet been fully elucidated. In this study, we systematically revealed the mechanism of action of AS in intervening against MASH through integrated transcriptomic and metabolomic analyses, coupled with molecular biological validation. These findings provide a scientific basis for the application of AS.

Integrated multiomics analysis serves as a powerful tool for deciphering the complex mechanisms of action of natural medicines. It enables a comprehensive elucidation of the dynamic responses of biological systems from two complementary perspectives: alterations in gene expression and changes in metabolite profiles. In the present study, correlation analysis of transcriptomic and metabolomic data uncovered the core regulatory network underlying AS-mediated MASH intervention. With respect to the correspondence between DEGs and differential metabolites, AS significantly modulated the expressions of 30 DEGs (e.g., *Gm15622*, *Pdia6*, *Derl3*, *Serpine1*, and *Il1a*) and 60 characteristic metabolites (encompassing bile acids, glycerophospholipids, fatty acid derivatives, and other categories). Notably, these differential molecules were collectively enriched in core pathways related to lipid metabolism and inflammatory response. For example, the *Gm15622* gene downregulated in the transcriptome, which is a key regulator promoting hepatic lipid accumulation, showed a positive correlation with the changes in fatty acid metabolite levels (e.g., heptadecanoic acid and 4-acetylbutyric acid) in the metabolome. By inhibiting *Gm15622* expression, AS effectively reversed the abnormal accumulation of fatty acids in the MASH model, which is consistent with the previously reported mechanism by which metformin ameliorates hepatic lipid deposition through *Gm15622* suppression (Ma et al., 2020). Meanwhile, the expression level of the AS-upregulated *Pdia6* gene, which is a key molecule possessing oxidoreductase activity, showed a positive correlation with the antioxidant-related metabolite levels (Huang et al., 2023). This observation indicates that *Pdia6* may alleviate oxidative stress and synergize with these metabolites to ameliorate hepatocellular injury.

At the pathway level, both transcriptomic and metabolomic data were co-enriched in key signaling pathways, including the AGE–RAGE pathway, glycerophospholipid metabolism, arachidonic acid metabolism, and the NF-κB pathway. For instance, in the AGE–RAGE pathway, the transcriptomic data detected abnormally high expression of genes such as *Il1α* and *Serpine1*, while the metabolomic data revealed dysregulated levels of metabolites, including adenosine diphosphate ribose and abscisic acid. Following AS treatment, not only was the overexpression of these genes significantly inhibited but also the abnormal levels of related metabolites were concurrently corrected. This formed a “gene-metabolite” synergistic regulatory network, thereby blocking oxidative stress and inflammatory responses mediated by this pathway. In addition, within the glycerophospholipid metabolism pathway, the changes in the expressions of genes such as *Scd1* and *Cyp2c55* in the transcriptome were closely associated with the adjustments of phospholipid metabolite levels (e.g., 25-acetylglucoside and 1-pyrroline-2-carboxylic acid) in the metabolome. These molecules collectively participate in the restoration of hepatic lipid homeostasis, further confirming the ability of of AS to synergistically regulate the pathological process of MASH through multitarget and multipathway actions.

Aberrant activation of the AGE–RAGE pathway is a key driving factor in MASH progression. Accumulating evidence has confirmed that the AGE–RAGE interaction can activate the NADPH oxidase–ROS signaling pathway, triggering excessive ROS production and concurrently promoting the release of pro-inflammatory cytokines (e.g., IL-1 and IL-6), thereby forming an “oxidative stress-inflammation” amplification loop (Wautier et al., 2001). In the MASH pathological microenvironment, the accumulation of AGEs not only directly induces oxidative stress but also activates downstream signaling cascades by binding to RAGE, thereby promoting the release of inflammatory factors, disrupting lipid metabolism, and causing hepatocellular injury (Asadipooya et al., 2019; Tilg and Moschen, 2010; Wang et al., 2021). In this study, we found that the protein expressions of AGE and RAGE in the liver of the HFD-/CCL_4_-induced MASH mice model were upregulated, accompanied by an increase in the oxidative stress marker MDA and a decrease in the activities of antioxidant enzymes SOD and GSH. These results confirm that the AGE–RAGE pathway is in an activated state in the MASH model.

After AS treatment, not only were the protein levels of AGE and RAGE reduced, blocking the activation of the AGE–RAGE pathway, but the abnormal expression levels of ferroptosis-related molecules were also concurrently reversed, leading to a decrease in hepatic Fe^2+^ accumulation. Furthermore, AS downregulated the expressions of key ferroptosis-promoting factors ALOX15 and ACSL4 while also upregulating those of the antioxidant proteins Nrf2 and HO-1, as well as the expressions of the ferroptosis-inhibitory proteins GPX4, FTH1, and SLC7A11, thereby restoring the activities of antioxidant enzymes. These findings indicate that by inhibiting the AGE–RAGE pathway, AS exerts a “pathway inhibition-dual protection” mode of action: on the one hand, it alleviates oxidative stress-induced injury, and on the other hand, it blocks the occurrence of ferroptosis. Existing studies have confirmed that ferroptosis plays a crucial role in the progression of hepatic fibrosis and inflammation in MASH (Zhang et al., 2024), and AGE–RAGE pathway-mediated oxidative stress is an important inducer of ferroptosis (Chen et al., 2024). Therefore, targeting this pathway is as a potential therapeutic strategy for MASH. This study is the first to confirm that AS exerts a protective effect through this mechanism, providing a novel therapeutic target for natural medicine-based intervention in MASH.

The JAK–STAT signaling pathway is a key signal transduction pathway that mediates inflammatory responses and plays a pivotal role in the inflammatory progression of MASH (Goswami and Kaplan, 2017). Under conditions of metabolic disorder and liver injury induced by HFD/CCL_4_, stimuli such as lipopolysaccharides derived from intestinal flora dysbiosis and inflammatory factors released by adipose tissue can activate the JAK1–STAT3 pathway. This activation promotes the phosphorylation of STAT3 and its translocation into the nucleus, initiating the transcription of pro-inflammatory factors such as TNF-α and IL-1β (Gao, 2012; Van Quickelberghe et al., 2018), which, in turn, exacerbates hepatocellular inflammation, ballooning degeneration, and hepatic fibrosis (Zhou et al., 2020). In the present study, the ratios of p-JAK1/JAK1 and p-STAT3/STAT3 were significantly elevated in the liver of the MASH model mice. Concurrently, the levels of TNF-α and IL-1β were increased in both the serum and liver tissues. Notably, these changes were significantly correlated with the decreased expression levels of GPX4 and SLC7A11, which are key negative regulators of ferroptosis. These findings confirm the regulatory role of the JAK–STAT pathway in ferroptosis. After AS treatment, the phosphorylation levels of JAK1 and STAT3 were significantly inhibited, the expressions of pro-inflammatory factors were reduced, and the pathological manifestations of inflammatory infiltration and fibrosis in the liver tissue were ameliorated. These results are consistent with the enrichment of the JAK–STAT pathway in the transcriptomic analysis, indicating that AS inhibits the transmission of inflammatory signals by blocking the activation of the JAK1–STAT3 pathway, thereby alleviating inflammatory injury in MASH. Notably, the JAK–STAT pathway and the AGE–RAGE pathway do not act independently. Instead, they form a cascade amplification effect via the “AGEs-RAGE-IL-6-JAK-STAT” axis, which continuously exacerbates oxidative stress and thereby provides the requisite conditions for the occurrence of ferroptosis. Existing studies have shown that the sustained activation of the JAK–STAT3 pathway is a key driving force for the progression of MASH from simple steatosis to hepatitis and fibrosis (Bernal et al., 2025), and inhibitors targeting this pathway have shown potential therapeutic prospects. This study confirms that AS can block this pathway through the effects of its natural metabolites, providing a novel natural medicinal option for the anti-inflammatory treatment of MASH.

Ferroptosis is an iron-dependent, non-apoptotic form of cell death characterized primarily by intracellular iron overload and excessive accumulation of lipid peroxides. It has been identified as a key pathogenic driver in the mechanism underlying the development of MASH. Clinical studies have demonstrated that hepatic iron overload is a common occurrence in MASH patients, and excess iron can accelerate disease progression by inducing ferroptosis (Liu et al., 2025; Zhang et al., 2025). The oxidative stress triggered by the activation of the AGE–RAGE and JAK–STAT pathways promotes ferroptosis through multiple mechanisms: on the one hand, excessive ROS can directly facilitate the release of iron ions from ferritin, exacerbating intracellular iron overload; on the other hand, ROS-mediated depletion of GSH impairs the antioxidant activity of GPX4, leading to massive accumulation of lipid peroxide products. This ultimately disrupts the integrity of hepatocyte membranes and induces cell death.

In the present study, concurrent abnormalities in lipid peroxide products, iron overload-related metabolites, and key molecules of the AGE–RAGE and JAK–STAT pathways in the MASH model—along with the coordinated reversal of these indices following AS intervention—conclusively confirm that the AGE–RAGE and JAK–STAT pathways form a critical link via oxidative stress, ultimately driving ferroptosis-mediated hepatocyte injury and constituting the core pathological axis of MASH progression. The elucidation of this mechanism integrates three previously independently studied pathological pathways into a complete “metabolic disorder-signal activation-oxidative stress-cell death” cascade, providing a novel perspective for understanding the pathogenesis of MASH.

This study also has certain limitations. Although the HFD/CCL_4_ mouse model can simulate the core pathological features of MASH, it cannot fully replicate the complex pathophysiological processes of human MASH. The experimental duration was relatively short, and thus, the long-term therapeutic effects of AS could not be evaluated. Daily food intake was not systematically measured throughout the study. The absence of these data precludes a definitive distinction between body weight changes resulting from direct metabolic improvements and those that are secondary to alterations in feeding behavior. Future research will focus on constructing animal models that more closely resemble human MASH, conducting long-term toxicity and efficacy studies, and verifying the functions of key targets through cellular experiments to provide more substantial evidence for the clinical translation of AS.

## 5 Conclusion

Our findings indicate that AS can inhibit the progression of MASH induced by HFD/CCL_4_ in mice. Through metabolomic and transcriptomic analyses, combined with experimental validation, we discovered that AS suppresses MASH progression by inhibiting the AGE–RAGE signaling pathway to reduce ferroptosis and by blocking the JAK1–STAT3 signaling pathway and attenuate inflammation, revealing its potential as a novel therapeutic strategy for MASH.

## Data Availability

The data presented in the study are deposited in the Mendeley data repository (https://data.mendeley.com/datasets/twvzcdrbz2/1), DOI 10.17632/twvzcdrbz2.1.

## Glossary

MASH: Metabolic dysfunction-associated steatohepatitis
AS: Aqueous extract of *Artemisia scoparia*
DCA-C: 4,5-dicaffeoylquinic acid
PCoA: Principal coordinate analysis
DEGs: Differentially expressed genes
LEfSe: Linear discriminant analysis effect size
GO: Gene Ontology
KEGG: Kyoto Encyclopedia of Genes and Genomes
PLS-DA: Partial least squares discriminant analysis
OPLS-DA: Orthogonal partial least squares discriminant analysis
QC: Quality control
ALT: Alanine aminotransferase
AST: Aspartate aminotransferase
CHO: Cholesterol
TG: Triglycerides
LDL-C: Low-density lipoprotein cholesterol
HDL-C: High-density lipoprotein cholesterol
AGE–RAGE: The advanced glycation end product–receptor for advanced glycation end products pathway
JAK–STAT: The Janus kinase–signal transducer and activator of transcription signaling pathway
NF-κB: The nuclear factor-kappa B pathway
IMR-TRP: Inflammatory mediator regulation of TRP channels
ALOX15: Arachidonate 15-lipoxygenase
ACSL4: Acyl-CoA synthetase long-chain family member 4
GPX4: Glutathione peroxidase 4
FTH1: Ferritin heavy chain 1
Nrf2: Nuclear factor erythroid 2-related factor 2
HO-1: Heme oxygenase-1
GAPDH: Glyceraldehyde-3-phosphate dehydrogenase
SLC7A11: Solute carrier family 7 member 11
DEMs: Differentially expressed metabolites
DEGs: Differentially expressed genes
