# Supplementary material for: Artemisia scoparia extract inhibits oxidative stress and ferroptosis to ameliorate MASH through AGE-RAGE and JAK1-STAT3 signaling
Source: Front Pharmacol. 2026 Apr 15;17:1792221. doi: 10.3389/fphar.2026.1792221 (PMC13125038; doi:10.3389/fphar.2026.1792221)
Supplement: Supplementary file 1 [file Supplementaryfile1.docx]

Antibody information

| Type | Target | Clone Type | Supplier | Cat or Cas | Dilution ratio |
| --- | --- | --- | --- | --- | --- |
| Primary antibody | Nrf2 | Rab mAb | Abcam, UK | ab137550 | 1:1000 |
|  | HO-1 | Ms mAb | Abcam, UK | ab13248 | 1:1000 |
|  | Alox15 | Rab pAb | Abcam, UK | ab80495 | 1:1000 |
|  | ACSL4 | Rab mAb | Abcam, UK | ab155282 | 1:1000 |
|  | SLC7A11 | Rab mAb | Abcam, UK | ab175186 | 1:1000 |
|  | GPX4 | Rab mAb | Abcam, UK | ab125066 | 1:1000 |
|  | FTH1 | Rab mAb | Abcam, UK | ab183781 | 1:1000 |
|  | AGE | Rab pAb | CST, China | C01328 | 1:1000 |
|  | RAGE | Rab mAb | Abcam, UK | ab216329 | 1:1000 |
|  | p-JAK1 | Rab pAb | Affinity, China | AF2012 | 1:1000 |
|  | JAK1 | Rab mAb | ABclonal, China | A18323 | 1:1000 |
|  | p-STAT3 | Rab mAb | Abcam, China | ab32143 | 1:1000 |
|  | STAT3 | Rab mAb | CST, China | 9139T | 1:1000 |
| Secondary antibody | Goat anti-rabbit IgG |  | Boster, China | BA1054 | 1:3000 |
|  | Goat anti-mouse IgG |  | Boster, China | BA1050 | 1:3000 |
